# Enhanced permittivity of negative permittivity middle-layer sandwich polymer matrix composites through conductive filling with flake MAX phase ceramics

**DOI:** 10.1039/d0ra03493b

**Published:** 2020-07-20

**Authors:** Zhuo Wang, Jiahao Fan, Xu Guo, Jiamin Ji, Zixiong Sun

**Affiliations:** School of Materials Science and Engineering, Shaanxi Key Laboratory of Green Preparation and Functionalization for Inorganic Materials, Shaanxi University of Science and Technology Xi’an 710021 People’s Republic of China wangzhuo@sust.edu.cn +86-15114845870; School of Electrical Informatica and Artificial Intelligence, Shaanxi University of Science and Technology Xi’an 710021 People’s Republic of China

## Abstract

Polymer matrix composites are expected to promote the development of embedded packaging technology for circuit boards, but it is still impossible to obtain polymer matrix composites with high permittivity and low loss tangent simultaneously. In this study, a laminated composite with a middle-layer possessing negative permittivity effects was prepared by hot pressing sintering using MAX phase ceramics as a conductive filler. High permittivity (170@1 kHz) and low loss tangent (0.3@1 kHz) were achieved in traditional sandwich polymer matrix composites (SPMCs). Its high permittivity can be explained by the series capacitor model and the interfacial polarization promoted by the flake structure of the MAX phase ceramics. Low loss tangent is guaranteed by the ohmic barrier effect caused by the huge resistance difference between adjacent layers in the composite material. These SPMCs with special structure are expected to provide new ideas for developing embedded capacitors.

## Abstract 2

In this study, a laminated composite with a middle-layer possessing negative permittivity effects was prepared by hot pressing sintering using MAX phase ceramics as a conductive filler.

## Graphical abstract

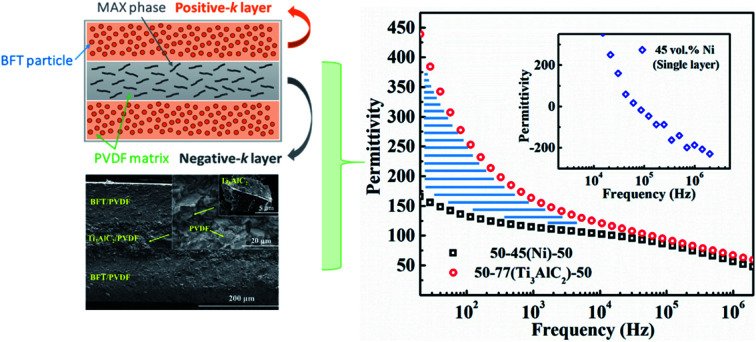

## 1 Introduction

The growing demand for the miniaturization of electronic devices and to make them intelligent has driven advances in electronic device packaging technology. Nevertheless, the traditional packaging technology cannot adapt to the current demand for electronic equipment and based on this a large number of researchers are committed to the development of new electronic device packaging technology. Embedded packaging technology changes passive components from traditional discrete surfaces to embedded substrates, reducing the thickness, volume and quality of the entire circuit board, thus further realizing the versatility and miniaturization of electronic equipment. In passive components, capacitors account for more than half of them, so the study of dielectric materials for embedded capacitors with excellent performance is the key to the commercialization of embedded technology.^1–3^

In recent years, polymer materials have received extensive attention because of their low processing temperature, high breakdown field strength, low cost and good compatibility with printed circuit substrates. However, the permittivity of single-component polymer material is generally very low and far away from practical applications. Therefore, by compounding the polymer with other materials, researchers have obtained high permittivity polymer based composites with improved dielectric properties.^4,5^ At present, there are about three kinds of technical routes to obtain high permittivity polymer matrix composites: (1) ceramic/polymer composites, in which the filler is the traditional giant permittivity ceramic powder.^6–8^ In this way, permittivity of the obtained composites can be increased to a certain extent on the basis of low loss tangent, but the degree of improvement depends too much on the filling ratio of the filler. High ceramic filler filling will lead to rapid deterioration of the loss tangent and mechanical properties of the composites.^9,10^ (2) Conductor/polymer composites and metals, in which graphene and related materials serve as conductive fillers.^11–13^ For conductor–polymer composites, although the permittivity may be very high, it is usually accompanied by very high loss tangent due to the tunneling effect or ohmic conduction.^14,15^ (3) Multilayer structural composites; interface polarization between adjacent layers of layered composite materials can effectively improve the permittivity.^16,17^ However, the increase in the permittivity of this structure depends too much on the number of layers of multilayer composites.^17^ At the same time, an excessive number of composite layers will make the preparation process more complex, resulting in increasing cost. Combining the above technical routes, on the basis of balancing the advantages and disadvantages of the existing polymer-based high permittivity materials, we designed laminated composite materials with a negative dielectric constant middle-layer. Finally, we expect to achieve a high dielectric constant and low loss tangent in traditional sandwich composites. It also provides a new research idea for the optimization of the dielectric properties of polymer matrix composites.

**Fig. 1:**
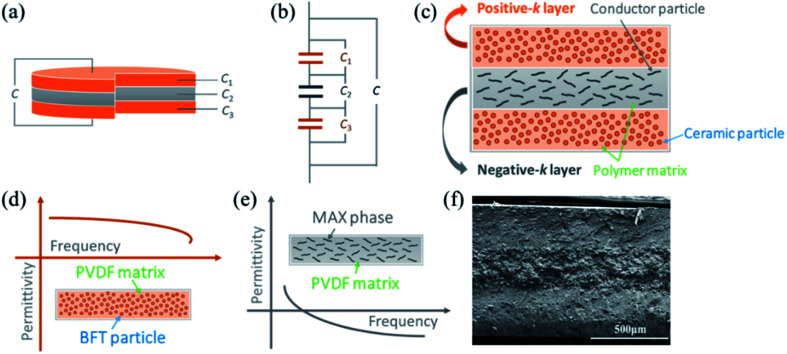
Schematic of sandwich-structured composites.

As shown in Fig. 1a and b, sandwich polymer matrix composites (SPMCs) can theoretically be equivalent to being concatenated by three capacitors. As we all know, when three capacitors are in series in a circuit, their total capacitance can be expressed as1
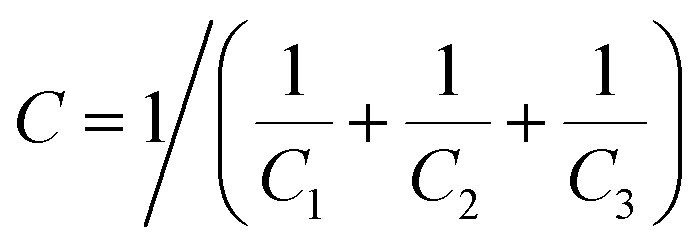
*C*_1_, *C*_2_ and *C*_3_ are the capacitance of each layer. Typically, the capacitance value of the material we are using is positive (*C*_1_, *C*_2_ and *C*_3_ are positive), so the *C* of the SPMCs will be smaller than any of them. However, if the middle-layer capacitor *C*_2_ is negative and the top-level capacitor *C*_1_ and the underlying capacitor *C*_3_ are still positive, the *C* of the SPMCs will improve significantly. It is particularly important that when the absolute values of (1/*C*_1_ = 1/*C*_3_) and 1/*C*_2_ are infinitely close, the theoretical maximum of *C* of SPMCs will be achieved. The conversion between capacitance permittivity and capacitance can be regarded as

2 *C* = *εS*/4π*kd*.

Based on this, we assume that the permittivity of SPMCs will be increased when the sandwich composite materials have a negative permittivity middle-layer and the absolute value of the negative permittivity is close to the positive permittivity of the upper and lower layers.

The structure used in this design is shown in Fig. 1c in which the top and bottom layers of the SPMCs are the positive permittivity layer. The positive dielectric constant layer is usually obtained by the compositing of a ceramic powder with high permittivity and a polymer.^18,19^ In this design, Ba(Fe_0.5_Ta_0.5_)O_3_ (BFT) ceramic powders with excellent dielectric properties and mature preparation technology are used as fillers for the positive permittivity layer.^20^ Because of the better dielectric properties than those of other traditional polymer matrix materials and the polar nature of the polymer, PVDF is selected as the polymer matrix material.^21^

The key to the high permittivity of the SPMCs in this design is the middle-layer with negative permittivity. Negative permittivity is usually considered as a special property of conductive materials.^22,23^ However, the negative permittivity absolute value of these materials is greater than 10^8^, which is not suitable for this design. Some studies have shown that the negative permittivity of conductive materials is affected by the effective concentration of free electrons in the materials.^22,24^ Therefore, a lower absolute negative permittivity middle-layer can be obtained by controlling the filling rate of conductive fillers in composite materials. In this design, the negative permittivity middle-layer adopts the M_*n*+1_AX_*n*_ (MAX) phase powder with excellent conductivity and thermal stability as the conductive filler. PVDF is used as the matrix to ensure the compatibility of adjacent layers. As a hotspot of material research in recent years, MAX phase materials have excellent properties of both metals and ceramics, so they are also called cermets.^25–31^ A MAX phase material is a conductor of electricity, for which room temperature resistivity generally ranges from 0.07–2 μΩ m. The resistivity of most MAX phase materials is lower than that of titanium.^31^ The conventional binary transition metal carbides and nitrides are less antioxidative in air at 500–800 °C, while the MAX phase material has strong antioxidative properties.^29,32^ And the density of the MAX phase material is closer to that of the polymer matrix than the metal, which makes the two phases more uniform and less likely to be deposited during mixing and pressing. At the same time, the special lamellar structure of the MAX phase material may cause the composites to exhibit unexpected performance. In this work, we selected Ti_3_AlC_2_ materials with mature preparation technology and excellent electrical properties in the MAX phase material system as the conductive filler which is suitable for the negative permittivity middle-layer.

## 2 Experimental section

### 2.1 Materials and chemicals

Ti_3_AlC_2_ powders (200 mesh, >99.5%) were purchased from Jilin 11 Technology Co., Ltd. Poly(vinylidene fluoride) (PVDF, *M*_w_ = 534 000) was supplied by Sigma-Aldrich Co., LLC. In our previous work, nano-BFT powders have been successfully prepared by the oxalate coprecipitation method.^20^

### 2.2 Preparation of Ti_3_AlC_2_/PVDF and Ba(Fe_0.5_Ta_0.5_)O_3_/PVDF mixtures

Anhydrous ethanol was added into Ti_3_AlC_2_ and PVDF powders and was mixed mechanically for 3 hours. Then the mixture was dried for 3 hours at 85 °C to obtain the required Ti_3_AlC_2_/PVDF homogeneous mixture. A uniform BFT/PVDF mixture was prepared by repeating the above steps.

### 2.3 Preparation of SPMCs

In this work, SPMCs were obtained through three steps. (1) BFT/PVDF mixture was pressed for 5 minutes at 20 °C and 4 MPa to form the edge layer (bottom layer) in the hot-pressing die. (2) Ti_3_AlC_2_/PVDF mixture was placed on the bottom layer and pressed for 5 minutes at 20 °C under 6 MPa to form an intermediate layer. (3) Above the middle-layer, BFT/PVDF mixture was pressurized for 20 minutes at 165 °C, then cooled to 20 °C to form SPMCs. The total thickness of SPMCs is 0.9 mm, and the thickness of each layer is 0.3 mm. In this design, the SPMCs are represented by the notation *m*–*n*–*m*, where *m* represents the volume percentage of BFT in the edge layer and *n* represents the volume percentage of Ti_3_AlC_2_ in the middle-layer.

### 2.4 Characterization methods

The XRD spectra of the samples were obtained by X-ray diffraction (XRD D/max-2200 pc). The microstructures of the SPMCs were evaluated by scanning electron microscopy (SEM, S-4800, Hitachi, Japan). Measurements of dielectric constant and loss of composites with frequency (20 Hz to 2 MHz) were undertaken using an Agilent-E4980A. Thermal gravity analysis (TG) and differential scanning calorimetry (DSC) were performed at a 10 °C min^−1^ heating rate using an STA409PC instrument in air.

## 3 Results and discussions

**Fig. 2:**
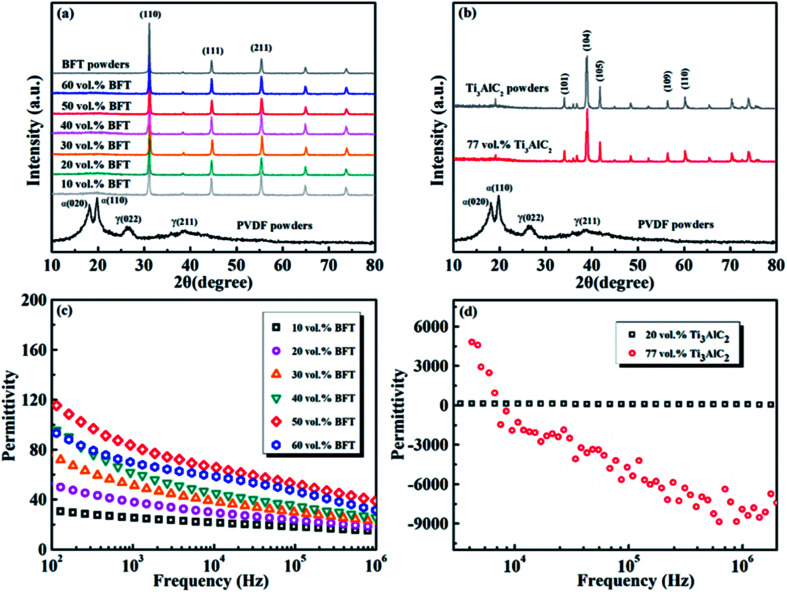
X-ray diffraction patterns of (a) BFT/PVDF and (b) Ti_3_AlC_2_/PVDF. Frequency dependence of permittivity for single layer (c) BFT/PVDF and (d) Ti_3_AlC_2_/PVDF.

Fig. 2a is the XRD pattern of the BFT\PVDF composite. From the diffraction pattern of PVDF, it can be concluded that PVDF is a semi-crystalline polymer mixed with α-crystal form and γ-crystal form. The sharp diffraction peaks appearing at 2*θ* = 18.3° and 19.9° correspond to the diffraction peaks of the (020) and (110) crystal planes of α-PVDF. The broadened diffraction peaks appearing at 2*θ* = 26.8° and 38.7° correspond to the diffraction peaks of the (022) and (211) crystal planes of γ-PVDF.^18^ In the XRD pattern of the BFT/PVDF composites, all the diffraction peaks corresponding to the BFT of cubic structure, and the diffraction peaks of PVDF flatten out, which are difficult to observe. The results can be attributed to the addition of a large proportion of BFT, which has medium concentration filling properties. When the volume fraction of filler is more than 20 vol%, the diffraction peak of BFT is very strong, which restrains the appearance of the diffraction peak of PVDF. The diffraction summit of PVDF is obviously weakened and not easy to observe. On the other hand, the BFT powder particles dispersing in the PVDF matrix destroy the regular structure of the PVDF molecular chain and increase the content of the amorphous phase of PVDF. The results show that the introduction of BFT powder into the composite can affect the crystal structure of the PVDF matrix. Fig. 2b shows the XRD diffraction pattern of 77 vol% Ti_3_AlC_2_/PVDF composites. The diffraction peaks at 34.0°, 39.0°, 41.8°, 56.6° and 60.3° degrees in the XRD pattern correspond to the (101), (104), (105), (109) and (110) planes of Ti_3_AlC_2_. It can be seen that in Ti_3_AlC_2_/PVDF composites, the corresponding diffraction peaks of PVDF have almost disappeared, and the corresponding diffraction peaks of Ti_3_AlC_2_ powder are stronger. The introduction of Ti_3_AlC_2_ powder with a high filling ratio in the PVDF matrix may destroy the orderly arrangement of PVDF molecular chains, resulting in the decrease in crystallinity and the increase in amorphous phase, so the corresponding diffraction peaks of PVDF gradually weaken. It can be seen in Fig. 2c that the permittivity increases with the increase in filling ratio for the BFT ceramic powders. When the BFT volume fraction is 50 vol%, the permittivity of the composites is located at 77 at 1 kHz, which can be attributed not only to the high permittivity of BFT, but also to the Maxwell–Wagner effect between ceramic nanoparticles and the polymer matrix.^33^ However, when the volume fraction of BFT is greater than 60 vol%, the permittivity of the composites decreases due to the introduction of low permittivity air into the ceramic filler.^34^Fig. 2d shows the frequency dependence of permittivity of single Ti_3_AlC_2_/PVDF composites. After continuous trial and research, when the filling rate of Ti_3_AlC_2_ reaches 77 vol%, the composite material changes from positive permittivity to negative permittivity. At this time, the absolute value of the negative permittivity of the composite materials is the smallest, which is exactly what this work needs.

**Fig. 3:**
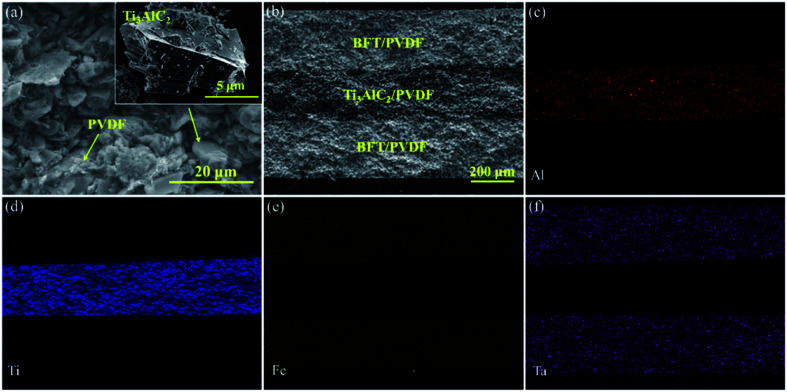
(a) SEM image of Ti_3_AlC_2_/PVDF composite (inset is SEM image of Ti_3_AlC_2_ powder). (b) SEM image of the fractured cross-section of SPMC. (c–f) EDS mapping of the fractured cross-section of SPMCs.

Fig. 3a presents the SEM image of a section of 77 vol% Ti_3_AlC_2_/PVDF composite. It can be seen that the PVDF matrix forms a continuous phase, and the lamellar Ti_3_AlC_2_ powder disperses in the PVDF matrix. Fig. 3b is the cross-section SEM image of the SPMC. The middle and darker layer represents the Ti_3_AlC_2_/PVDF negative permittivity layer, and the lighter upper and lower layers are the BFT/PVDF positive permittivity layers. The thickness of each layer is about 0.3 mm, and the interfaces between adjacent layers are clear. Fig. 3c–f are the EDS diagrams corresponding to Fig. 3b. It can be seen that the Ti and Al in Ti_3_AlC_2_ are evenly distributed in the middle-layer, the Fe and Ta in BFT are evenly distributed in the upper layer and the lower layer, which indicates that the SPMC required for the work is successfully prepared.

**Fig. 4:**
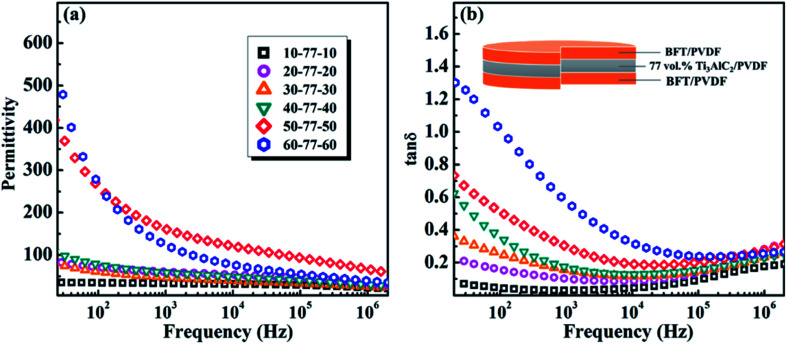
The frequency dependence of the (a) permittivity and (b) loss tangent of the negative permittivity middle-layer SPMCs with Ti_3_AlC_2_ as conductive fillers at room temperature.

Fig. 4 is the frequency dependence of the permittivity and loss tangent of the negative permittivity middle-layer SPMCs with Ti_3_AlC_2_ as the conductive filler. It can be seen that permittivity of the composites increases in turn with the increase of BFT ceramic powder in the edge layer. When the BFT content is 50 vol%, the permittivity of the composites reaches 170, which is 20 times that of pure PVDF. The exciting results are mainly due to the negative permittivity middle-layer of 77 vol% Ti_3_AlC_2_/PVDF. In the three capacitors series circuit equivalent to SPMCs, the capacitance value of one capacitor becomes negative, which leads to a sharp increase in the capacitance value of the whole circuit. In addition, the giant permittivity effect of BFT itself and the interfacial polarization between adjacent layers also contribute a part. Due to the large filling ratio of the conductive filler in the middle-layer, conductivity of the filler is much higher than that of the BFT/PVDF composite edge layer. When an electric field is applied to the material, charges in the material will accumulate between the adjacent layers and result in great differences in conductivity, which hinders the long-range movement of the charges, thus achieving the purpose of increasing the permittivity. When the proportion of BFT is more than 50 vol%, permittivity of the composites decreases sharply. This is because the excessive filling ratio will cause air, which has a lower permittivity, to enter the composites in large quantities and produce more defects, which will ultimately lead to the deterioration of the dielectric properties of the composites. For all these composites, frequency dependence of the dielectric constant can be obviously detected, and the decrease in permittivity with increasing frequency at lower frequencies is attributed to the seceding of interfacial polarization (MWS polarization effect). Because dipole orientation polarization and interfacial polarization work together on the dielectric properties of materials at low frequencies, and the overlap of polarization mechanisms will lead to the increase of loss tangent, composite materials exhibit relatively high loss tangent at low frequencies. At higher frequency, the loss tangent of the composites increases slowly, which is due to the dipole orientation polarization in the matrix PVDF. Since the large difference in resistance at the interface hinders the movement of the charge, when the BFT filling ratio is 50 vol%, the loss tangent of the SPMCs with the negative permittivity middle-layer at 1 kHz is maintained at about 0.3. Such a loss tangent is acceptable under the premise that the permittivity is increased so much.

**Fig. 5:**
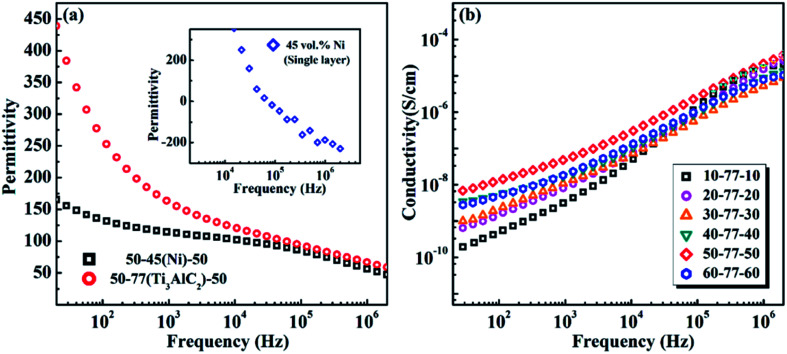
(a) The frequency dependence of the permittivity of the negative permittivity middle-layer SPMCs with Ni and Ti_3_AlC_2_ as conductive fillers at room temperature (the inset is the frequency dependence of the permittivity of a single layer Ni/PVDF composite). (b) Frequency dependence of conductivity of SPMCs with a negative permittivity middle-layer of Ti_3_AlC_2_ as conductive filler.

Fig. 5a displays the frequency dependence of the permittivity of the negative permittivity middle-layer SPMCs with intermediate layers made of different conductive materials as conductive fillers. The single layer Ni/PVDF composite exhibits a negative permittivity when the filling ratio is 45 vol%. The permittivities of negative permittivity middle-layer composites with two different conductive fillers are compared with the premise of keeping the edge layer 50 vol% BFT/PVDF unchanged. It can be seen from Fig. 5a that the permittivity of the composite material with Ti_3_AlC_2_ as the conductive filler is much larger than that of the composite material with Ni as the conductive filler. The unique flake structure of Ti_3_AlC_2_ provides an unexpected contribution to the improvement of the permittivity in the composite. It can be seen that the filling ratio of Ti_3_AlC_2_ is much higher than that of Ni and the unique special flake structure offers more interfaces in the negative permittivity middle-layer. The permittivity of composites is greatly affected by interfacial polarization at low frequencies, so the permittivity of composites with Ti_3_AlC_2_ as the conductive filler is much larger than that of composites with nickel as the conductive filler at low frequencies. The permittivities of the two SPMCs tend to be the same at high frequencies, which is due to the polarization mechanism of the composites changing from interfacial polarization to dipole polarization.

Fig. 5b displays the frequency dependence of the conductivity of SPMCs with a negative permittivity middle-layer. It can be observed that although the addition of Ti_3_AlC_2_ in the middle-layer has reached 77 vol%, the overall conductivity of the composite is still very small. This illustrates that the interface between the edge layer and the phase layer exhibits inhibition effects on the movement of charges and keeps the material with a larger resistance.^35,36^ The change trend of the conductivity of the composites with different BFT contents is consistent with permittivity. At 100 Hz, when the BFT content is 50 vol%, the conductivity of the material reaches 10^−8^ S cm^−1^, and the material still presents good insulation.

**Fig. 6:**
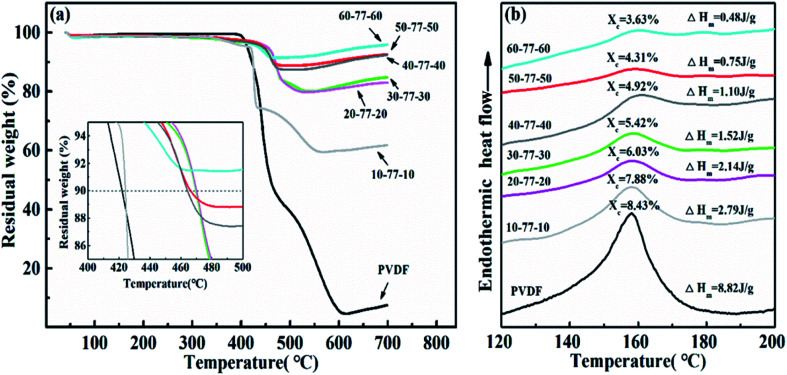
(a) TG curves and (b) DSC curves of SPMCs with a negative permittivity middle-layer of Ti_3_AlC_2_ as conductive filler.

Fig. 6 demonstrates the TG and DSC curves of SPMCs with a negative permittivity middle-layer. From Fig. 6a, it is revealed that the mass decomposition curves of SPMCs show the same trend as that of PVDF in the test temperature range, indicating that the introduction of BFT and Ti_3_AlC_2_ powder will not change the thermal decomposition mechanism of the PVDF matrix. Compared with the TG curve of PVDF, the initial decomposition temperature of SPMCs with a negative permittivity middle-layer shifts to high temperature, and the composites exhibit a higher initial decomposition temperature. When the mass loss of PVDF is 10%, the temperature is 422 °C, and then reaches 465 °C when the BFT filling ratio is more than 20 vol%. Negative permittivity middle-layer SPMCs exhibit better temperature stability, which can be attributed to uniformly distributed microstructures and the inherent properties of the fillers themselves. At the same time, BFT and Ti_3_AlC_2_ particles as barriers hinder the generation and removal of volatile by-products during thermal decomposition.

It is obtained from Fig. 6b that the crystallization temperature of PVDF is 158.7 °C. After the introduction of BFT and Ti_3_AlC_2_ powders, the crystallization temperature of the SPMCs moves toward high temperature, indicating that the fillers affect the crystallization behavior of PVDF, which is consistent with the XRD analysis as mentioned above. In order to further accurately study the influence of the introduced filler on the crystallization behavior of the polymer matrix, the crystallinity of the SPMCs is calculated by enthalpy change. The calculation formula of crystallinity is as follows:

3 *X*_c_ = Δ*H*_m_/*F*Δ*H*^∞^_m_

Δ*H*_m_ is the melting enthalpy (J g^−1^) of the composites. Δ*H*^∞^_m_ is the melting enthalpy of PVDF 100% crystallization, the value of which is 104.7 J g^−1^. *F* is the mass percentage of PVDF. The main factor that reduces the crystallinity of the composites is the destruction of the crystallization law in the PVDF molecular chain by the introduction of BFT filler powder. As a result, the crystallinity of the polymer is not complete and the crystallinity is reduced. The amount of filler added in this work belongs to the range of medium–high proportion filling, so the crystallinity of the material is greatly affected. Therefore, the crystallinity of the composites decreases with the increase in BFT volume fraction.

## 4. Conclusions

Based on the series capacitor model, a new structure of stacked positive permittivity edge layers and negative permittivity middle-layer was adopted. The composite material with high permittivity and low loss tangent was obtained by hot pressing sintering technique with Ti_3_AlC_2_ as the conductive filler and BFT as the high permittivity ceramic filler. Composites with high filling ratio flake Ti_3_AlC_2_ ceramic powders greatly increase the permittivity of SPMCs due to the increased amount of interfaces. Finally, the permittivity of the SPMCs increases to 170@1 kHz. The loss tangent of the material is kept at 0.3@1 kHz owing to the huge conductivity difference between adjacent layers, and the conductivity of the material remains at only 10^−8^ S cm^−1^. The thermal stability of the composites has been also greatly improved because of the introduction of BFT and Ti_3_AlC_2_, and all of these are expected to provide a new paradigm for the research of embedded capacitors.

## Conflicts of interest

There are no conflicts to declare.

## Supplementary Material
